# Inflammatory Bowel Disease: Immune Function, Tissue Fibrosis and Current Therapies

**DOI:** 10.3390/ijms25126416

**Published:** 2024-06-11

**Authors:** Jesús Cosín-Roger

**Affiliations:** 1 Departamento de Farmacología, Facultad de Medicina y Odontología, Universidad de Valencia, 46010 Valencia, Spain; jesus.cosin@uv.es; 2 CIBERehd (Centro de Investigación Biomédica en Red de Enfermedades Hepáticas y Digestivas), Instituto de Salud Carlos III, Monforte de Lemos, 3-5, 28029 Madrid, Spain

Inflammatory Bowel Disease (IBD) is a complex and challenging health problem that exerts a significant impact on the quality of life of millions of individuals worldwide [1]. Although IBD comprises Ulcerative Colitis (UC) and Crohn’s Disease (CD), they present several differences [2,3]. On the one hand, UC is characterized by a mucosal inflammation that usually starts in the rectum and can spread to other parts of the colon without affecting the rest of the gastrointestinal tract. UC patients present intestines with a reduced number of intestinal crypts, which are also disrupted, and this is associated with increased cellular infiltration of macrophages, lymphocytes, granulocytes, and plasma cells. These patients experience symptoms such as bloody diarrhoea, abdominal pain, rectal bleeding, weight loss, fever and tenesmus, which lead to more severe phenotypes including epithelial dysplasia, toxic megacolon, or even colon carcinoma [4,5]. On the other hand, CD is characterized by transmural, segmental, and asymmetrical inflammation that can appear in any segment of the gastrointestinal tract from the mouth to the anus, although the colon and ileum are the most frequently affected areas. Patients exhibit intestines with disrupted intestinal crypts, transmural inflammatory infiltrates, and lymphoid aggregates. The most common symptoms of this disease are chronic and bloody diarrhoea, abdominal pain, fatigue, weight loss, and rectal bleeding. During their lives, half of those with CD develop extraintestinal manifestations in other parts of the body, such as the eyes, skin, and joints, and suffer complications, such as strictures and fistulas [6,7,8].

One of the most serious complications of both CD and UC, although to a lesser extent in the latter case, is intestinal fibrosis, a condition characterized by an excessive deposition of extracellular matrix components leading to tissue scarring and stricture formation [9,10]. Diagnosis of this IBD-associated complication is still not well defined, since it often occurs concurrently with inflammation and may not be easily distinguishable during imaging or endoscopic evaluation [11,12,13]. Moreover, the clinical utility of the serum levels of some biomarkers proposed for assessing intestinal fibrosis, such as matrix metalloproteinases (MMPs) and their inhibitors (TIMPs), is unclear [14,15]. Therefore, understanding the molecular mechanisms underlying the development of this complication continues to be an important goal, which is crucial to the development of targeted therapies to prevent or reverse it.

In recent years, research has shed light on the intricate interplay between chronic inflammation and fibrosis within the pathogenesis of IBD [16,17]. In fact, inflammation is a key driver of fibrotic processes in the intestine, with pro-inflammatory cytokines such as interleukin-1β (IL-1β), interleukin-6 (IL-6), and tumour necrosis factor-alpha (TNF-α) playing a pivotal role in promoting fibroblast activation and collagen production [18,19,20]. In addition, the dysregulated immune response present in IBD patients leads to sustained inflammation, which in turn triggers fibroblast proliferation and differentiation into myofibroblasts, the primary effector cells responsible for collagen deposition and tissue remodelling in fibrotic lesions [21].

The treatment of IBD patients, including those with UC and CD, is determined by several parameters such as the disease activity, location, and behaviour. It is important to note the primary goals of current therapeutic strategies: (a) to induce and prolong remission periods; (b) to improve the quality of life of IBD patients; and (c) to prevent complications and disability [22]. The ongoing therapeutic alternatives for these patients include a wide range of drugs which can be classified in the following two groups: immunomodulators and biologics such as anti-tumour necrosis factor alpha (TNF-α) agents, anti-integrins, and anti-interleukins (IL-12/23) and novel and emerging therapies for patients who have not responded to traditional treatments and exhibit moderate-to-severe disease such as subcutaneous and oral anti-TNFα preparations, antiadhesion agents, cytokine inhibitors, Janus kinase (JAK) inhibitors, phosphodiesterase (PDE) inhibitors, sphingosine-1 phosphate (S1P) receptor modulators, and micro-RNA-124 (miR-124) up regulators [23,24,25]. Despite the progress made to date with the pharmacological treatment of IBD and the notable improvement achieved in the quality of life of patients, there is still a gap that needs to be bridged if a drug that allows a complete remission of the disease is to be developed.

Current therapeutic strategies to combat intestinal fibrosis are limited, since anti-inflammatory and immunosuppressive strategies often fail to prevent or reverse this complication. Moreover, there is a lack of translation of recent experimental results into the development of new drugs, while there continues to be a lack of consensus defining clinical trial endpoints [26]. In fact, the only way to remove fibrotic tissue at the present date is surgical intervention involving resection or strictureplasty. However, patients undergoing such interventions present a high incidence of relapse, and the surgery can strongly impact their quality of life [27,28,29]. Hence, there has been an increased interest in recent years in developing new drugs that target fibrotic molecules in order to prevent or even revert this complication in a way that allows surgery to be avoided. These potential pharmacological approaches include inhibitors of the most relevant profibrotic signalling pathways, such as the TGF-β and PDGF pathways, as well as agents that modulate the activity of the main protagonists of intestinal fibrosis, namely, fibroblasts and myofibroblasts [30,31]. However, further research is essential in order to identify the most effective and safest anti-fibrotic therapies for IBD patients.

Moreover, recent studies have highlighted the role of the gut microbiota in modulating the inflammatory and fibrotic responses in IBD [32,33]. Dysbiosis, characterized by an imbalance in the composition and function of the gut microbiota, has been widely associated with the pathogenesis of IBD and the development of intestinal fibrosis. Specific microbial species have been shown to promote inflammation and fibrosis in the gut through the production of pro-inflammatory metabolites and the modulation of immune responses [34,35,36,37]. Based on this evidence, targeting the gut microbiota through probiotics, prebiotics, or faecal microbiota transplantation represents a promising approach to modulate the inflammatory and fibrotic processes in IBD and prevent the progression to intestinal fibrosis.

In conclusion, the relationship between inflammation and fibrosis in IBD is a complex and multifaceted process that involves immune dysregulation, microbial dysbiosis, genetic susceptibility, and environmental factors. Understanding the molecular mechanisms driving intestinal inflammation and fibrosis in IBD is essential to the development of targeted therapies to improve long-term outcomes in IBD patients. In this Special Issue, potential new pharmacological targets and serum biomarkers of IBD are proposed, although they require further validation by future studies. Moreover, different methods have been compared in order to monitor one of the most common drugs used in IBD patients, infliximab. Finally, the current knowledge about the pathophysiology of IBD, focusing on the innate immune system and the role of CD4 T-cells, has also been reviewed.
